# Agreement between results of meta-analyses from case reports and clinical studies, regarding efficacy and safety of idursulfase therapy in patients with mucopolysaccharidosis type II (MPS-II). A new tool for evidence-based medicine in rare diseases

**DOI:** 10.1186/s13023-019-1202-6

**Published:** 2019-10-21

**Authors:** Miguel Sampayo-Cordero, Bernat Miguel-Huguet, Almudena Pardo-Mateos, Andrea Malfettone, José Pérez-García, Antonio Llombart-Cussac, Javier Cortés, Marc Moltó-Abad, Cecilia Muñoz-Delgado, Marta Pérez-Quintana, Jordi Pérez-López

**Affiliations:** 1 Medica Scientia Innovation Research (MedSIR), Rambla de Catalunya 2, 2D, 08007 Barcelona, Spain; 2 Colorectal Unit, Department of Surgery, Hospital de Bellvitge, Barcelona, Spain; 3 Albiotech consultores y redacción científica S.L., Madrid, Spain; 4 IOB, Institute of Oncology, QuironSalud Group, Madrid & Barcelona, Spain; 5 FISABIO - Hospital Arnau de Vilanova, Valencia, Spain; 6 Unit of Rare Diseases, Hospital Vall d’Hebron, Barcelona, Spain; 7 Department of Internal Medicine, Hospital Gregorio Marañón, Madrid, Spain; 8 Department of Internal Medicine, Hospital San Juan de Dios, Aljarafe, Seville, Spain

**Keywords:** Systematic review, Meta-analysis, Case reports, Clinical studies, Mucopolysaccharidosis type II, Enzyme replacement therapy, Evidence-based medicine

## Abstract

**Background:**

A preliminary exploratory study shows solid agreement between the results of case reports and clinical study meta-analyses in mucopolysaccharidosis Type I (MPS-I) adult patients. The aim of the present study is to confirm previous results in another patient population, suffering from mucopolysaccharidosis Type II (MPS-II).

**Methods:**

A systematic review and meta-analysis of case reports published by April 2018 was conducted for MPS-II patients treated with enzyme replacement therapy (ERT). The study is reported in accordance with PRISMA and MOOSE guidelines (PROSPERO database code CRD42018093408). The assessed population and outcomes were the same as previously analyzed in a meta-analysis of MPS-II clinical studies. The primary endpoint was the percent of clinical cases showing improvement in efficacy outcome, or no harm in safety outcome after ERT initiation. A restrictive procedure to aggregate case reports, by selecting standardized and well-defined outcomes, was proposed. Different sensitivity analyses were able to evaluate the robustness of results.

**Results:**

Every outcome classified as “acceptable evidence group” in our case report meta-analysis had been graded as “moderate strength of evidence” in the aforementioned meta-analysis of clinical studies. Sensitivity, specificity, and positive-negative predictive values for results of both meta-analyses reached 100%, and were deemed equivalent.

**Conclusions:**

Aggregating case reports quantitatively, rather than analyzing them qualitatively, may improve conclusions in rare diseases and personalized medicine. Additionally, we propose some methods to evaluate publication bias and heterogeneity of the included studies in a meta-analysis of case reports.

## Background

The low prevalence of rare diseases, the phenotype heterogeneity and the long latency period, may prevent and/or make the possibility of performing randomized clinical trials (RCTs) and large studies extremely difficult [1, 2]. Therefore, with these diseases, knowledge of treatment efficacy or any other type of clinical knowledge must be based only on observational studies, rare disease registries and case reports [2], where real world data and evidence play an important role in health care decisions [3]. However, RCTs are assessed, in evidence-based medicine as the best corroboration of the efficacy of new treatments, while case reports show a lower grade level of evidence [1].

Previous FDA drug approvals with breakthrough status suggest that sometimes non-controlled studies can provide the same quality of evidence to demonstrate a positive risk–benefit ratio as individual RCTs [2, 4]. Accordingly, randomized phase II controlled-trials were not superior to single-arm phase II trials in predicting phase III study success [5].

In rare diseases, research based on registries and case studies is likely the best option, due to lack of patients, and case reports are often the primary evidence of the effectiveness of a new therapy or treatment [6]. Due to prior considerations, an increasing interest in case report analyses, and combining their results in systematic reviews, exists [7, 8].

Case report databases are developed as the Consensus-based Clinical Case Reporting (CARE) guidelines [9] attempt to homogenize and upgrade the quality of the information published in case reports; however, there are still questions about how to aggregate them in ways that would be most meaningful [8].

A previous systematic review of clinical studies, evaluating the effectiveness of enzyme replacement therapy (ERT) in adults (≥ 18 years) with mucopolysaccharidosis Type I (MPS-I), rated the strength of evidence (SOE) for ERT on each outcome with the Grading of Recommendations Assessment, Development and Evaluation (GRADE) criteria [10]. Another study showed a good rate of agreement between SOE and specific outcomes in a case report meta-analysis and clinical study meta-analyses [11]. This agreement has not been confirmed in other patient populations. Furthermore, the mentioned case report meta-analysis and clinical study meta-analysis were developed by the same research group.

Our proposal performed a meta-analysis of case reports of MPS-II patients treated with ERT, and compared the degree of evidence assigned to each outcome, vs. what was assigned in a previous clinical study meta-analysis, published by an independent research group. In a population suffering from MPS-II, we sought to confirm the impressive rate of agreement seen between case reports and clinical study meta-analyses in patients with MPS-I [11].

## Methods

### Data sources and study selection

A systematic review of case reports published through April 2018 was conducted for MPS-II patients treated with ERT. It was carried out on EMBASE, MEDLINE, The Cochrane Library (Cochrane Database of Systematic Reviews, Cochrane Central Register of Controlled Trials), Cochrane Methodology Register and Health Technology Assessment Databases) as well as on the Latin American and Caribbean Literature on Health Sciences (LILACS). The search strategy retrieved citations in databases containing the subject headings: Hunter syndrome, enzyme replacement, iduronate 2 sultatase, idursulfase, case report, case study, and medical record review. The search terms were adapted and used with different bibliographic databases (see Additional file 1: Table S1). An inclusive approach was used for low disease incidence. We included all-language articles/documents addressing one or more key questions, associated interventions and outcomes. Selected abstracts and articles published in languages other than English were translated into English by native speakers. Study designs included case reports and reviews of these case reports, which were put into narrative form. Prospective and retrospective studies that aggregated patient data were not considered, although the individual data of each patient could be extracted. The same outcomes and populations analyzed in the meta-analysis of clinical studies, published by Bradley et al. [12], were evaluated. Bradley et al. had conducted a systematic review of randomized controlled trials, nonrandomized trials, observational studies, registry data, systematic evidence reviews, and health technology assessments (through December 31st, 2015).

Study subjects were males with enzymatically-confirmed MPS-II, of any age, phenotype, genotype, stage of progression, or family history. An intervention of interest was the intravenous administration of idursulfase.

### Quality assessment

The study was prospectively designed to confirm MPS-II patients, as well as results of agreement, which were observed in the MPS-I population [11]. The current meta-analysis is reported in accordance with the Preferred Reporting Items for Systematic Reviews and Meta-Analysis (PRISMA) and Meta-Analyses and Systematic Reviews of Observational Studies (MOOSE) guidelines [13, 14]. The protocol was published in the International Prospective Register of Systematic Reviews (PROSPERO) database (Code 42018093408).

Two investigators (SCM and PMA) entered findings into a database, independently reviewed citations/abstracts from the database and hand searches, and selected full relevant articles and documents for data extraction using preset criteria. Discrepancies were resolved through discussion or input from a third reviewer (PLJ).

### Previous study outcomes

In the meta-analysis of MPS-II clinical studies by Bradley et al. [12], each outcome had been scored with a SOE grade (high, moderate, low, and insufficient) based on the results of previous clinical studies [15]. Different outcomes could be classified based in their evidence level in two groups: Acceptable (high to moderate SOE grade) and unacceptable (low to insufficient SOE grade). Outcomes and SOE assigned were:

### Acceptable level of evidence

- Urinary glycosaminoglycans (uGAGs) level (μg/mg creatinine) reduction -- moderate.
- Liver volume -- moderate.
- Harm: development of antibodies -- moderate.

### Unacceptable level of evidence

- Harm: Rate of IRRs (infusion-related reactions) and SAEs (serious adverse events) -- low.
- 6-min walk test in meters (6MWT) -- low.
- Growth: height -- low.
- Pulmonary function (forced vital capacity (FVC%), normalized for age and sex --low.
- Joint range of motion (JROM) -- insufficient.
- Benefit and harm: physical disability/quality of life -- insufficient.
- Cardiac function -- insufficient.
- Sleep apnea -- insufficient.

The long-term outcomes were not ranked with SOE [12]. Authors said: “no studies addressed longer-term measurable ERT outcomes,” which they classified as “none.”

### Primary endpoint

Our meta-analysis of case reports considered the same outcomes analyzed in Bradley’s study [12]. We also scored each outcome based only on the results of previous narrative case reports. We divided the number of case reports with a modification for a specific outcome after ERT by the total number of case reports analyzed, and then measured as a percentage. It is important to consider that we could define an efficacy outcome as improved in a case report only if: (1) the method of outcome evaluation was described (Ex: An abdominal ultrasound to characterize the liver size was performed prior to the start of treatment, then at 6 months and 15 months), or (2) a quantitative measure was reported in the clinical case (Ex: urine GAGs declined to 12.17–26.1 mg/mmol creatinine). Only infusion-related reactions (IRR) that caused ERT dose changes were considered relevant. This restrictive procedure to define an outcome as improved (efficacy) or worsened (IRR) was considered a strong confirmatory method. Given this score, we assumed that the greater the percentage of case reports showing improvements or impairments (IRRs) for a specific outcome, the higher the grade of evidence for ERT-driven outcome modifications.

### Secondary endpoint

For secondary endpoints, the improvement in ERT was weakly defined; we assumed the existence of improvement with the mention of improvement in the case report (weak confirmatory method).

### Statistical methods

The primary endpoint was the percentage of case reports with an outcome modification after ERT, based on a strong confirmatory method. This percentage was calculated for each of the 11 outcomes analyzed. Additionally, we classified these outcomes in two groups (acceptable or unacceptable evidence), based on the percentage of case reports. To classify them, we tested the null hypothesis for the percentage of case reports with an outcome modification lower or equal to 5% (H0). We based our analysis on a one-sided binomial test. Multiplicity issues, derived from analyzing 11 outcomes [16] were adjusted by the step-up Benjamini-Hochberg procedure for a false discovery rate (FDR) of 10%, although more elaborate extensions of this method are used to assess the importance of the endpoints analyzed [17]. We classified a specific outcome as acceptable evidence if its *p*-value was equal to or lower than the FDR 10% critical value, which was calculated by ranking the outcomes from the lowest to the highest p-value. The FDR critical value is defined as the rank (r) divided by number of outcomes (nr), and multiplied by the accepted 10% rate of false discoveries. Outcomes not achieving this were classified as unacceptable evidence.

We evaluated the concordance between outcomes in the acceptable evidence group, based on the primary outcome and outcomes classified as high to moderate SOE in the Bradley meta-analysis [12]. The validity indices reported were sensitivity, specificity and predictive values, estimated with 95% confidence intervals (CIs).

The primary analysis was performed in the primary analysis set, which was considered in all case reports of MPS-II patients, treated with ERT with reported efficacy and safety. These case reports were written in narrative form (results were not aggregated) and published prior to the bibliographic search by Bradley et al. [12].

The secondary analysis evaluated concordance between outcomes in the acceptable evidence group, with a weak confirmatory assumption, and outcomes classified as high to moderate SOE in the Bradley meta-analysis [12]. Validity indices were also reported.

Secondary analysis evaluated the relative agreement between number of case reports showing a modification in a specific outcome and the SOE score (1 as insufficient; 2 as low; 3 as moderate, and 4 as high) reported from the clinical study meta-analysis. Spearman rank correlation was used to evaluate relative agreement. Results were presented in a scatter plot.

Sensitivity analysis was planned in three ways. First, we compared the measures of validity and relative agreement with strong and weak confirmatory methods in the primary analysis set. Second, we estimated measures of validity and relative agreement derived with a strong confirmatory assumption in three analysis sets:

1. Including all case reports, even those published after the bibliographic search by Bradley et al. (31 December 2015) [12].
2. Excluding all studies that had been analyzed in the Bradley study.
3. Excluding all congress communications.

We compared the validity indices based on different assumptions about the futility boundary (null hypothesis): 5% primary analysis-, 1, 10, 15, and 20%. The analyses were performed in the primary analysis set.

## Results

**Fig. 1:**
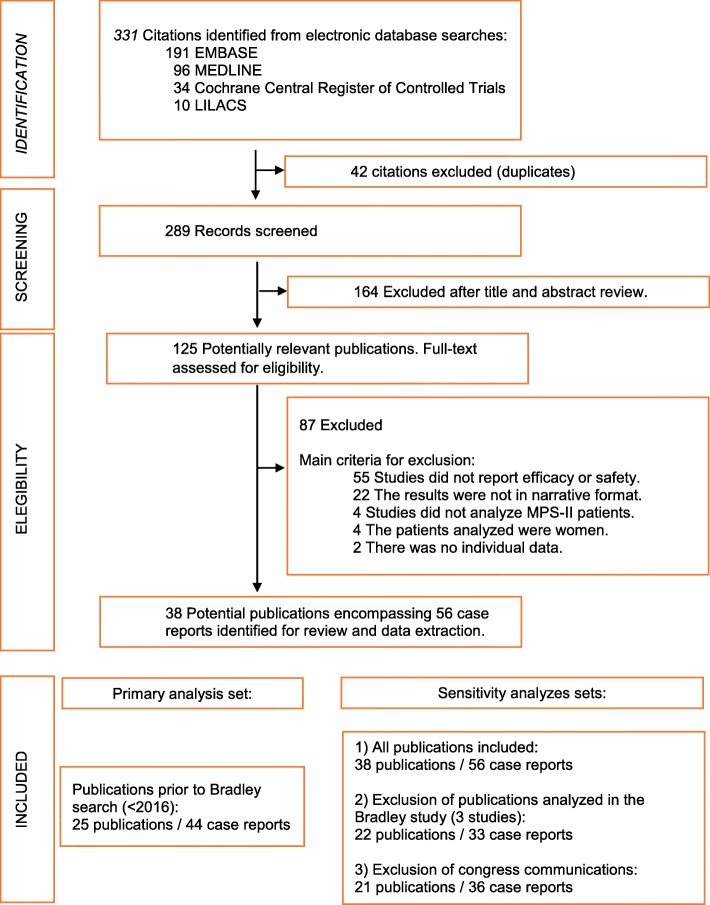
Flow diagram of case reports of patients with MPS-II published between January 2008 to April 2018

Database searches through April 13th, 2018 identified 331 citations and 289 unique abstracts. The reference of all abstracts screened and the reason for exclusion are reported in the Additional file 2. Out of 125 communications with a full text review, 38 articles and congress communications described a total of 56 case reports, and met inclusion criteria. Finally, 44 single-cases were considered for the primary set of analysis and 56 single-cases were analyzed in sensitivity analyses (Fig. 1). The characteristics of the 56 case reports are described in Additional file 1: Tables S2 and S3.

### Primary endpoint analysis

Table 1 describes the number of case reports that show a modification in a specific outcome after ERT, as defined by the strong confirmatory criterion, among the total number of case reports selected in the primary analysis set. Specific outcomes defined as modified in each case report are summarized in Additional file 1: Tables S2 and S3.

**Table 1:** Analysis of number of case reports showing improvement or impairment associated with ERT in a specific outcome, based on the strong confirmatory method

RANK	Outcomes & (SOE score*)	Nr. [+] / Total cases	*p*-value**	FDR 10% Critical value***	Evidence group****
1	uGAGs **(Moderate)**	20/44	< 0.0001	0.009	Acceptable
2	Liver V **(Moderate)**	8/44	0.001	0.018	Acceptable
3	Antibodies **(Moderate)**	6/44	0.022	0.027	Acceptable
4	6MWT (Low)	4/44	0.177	> 0.036	Unacceptable
4	JROM (Insufficient)	4/44	0.177	> 0.036	Unacceptable
5	Growth height (Low)	3/44	0.379	> 0.045	Unacceptable
6	IRR (Low)	2/44	0.653	> 0.055	Unacceptable
7	Pulmonary function (Low)	1/44	0.895	> 0.064	Unacceptable
8	Cardiac (Insufficient)	0/44	1	> 0.073	Unacceptable
8	QoL (Insufficient)	0/44	1	> 0.073	Unacceptable
8	Sleep apnea (Insufficient)	0/44	1	> 0.073	Unacceptable

Legend:The improvement is defined in accordance with the strong confirmatory method. The impairment was declared when an Infusion Related Reaction (IRR) caused a change in ERT dose.

*6MWT* 6-min walk test, *FDR* False discovery rate (Benjamini-Hochberg procedure), *IRR* infusion-related reaction, *JROM* Joint range of motion, *Nr. [+]* Number of case reports showing improvement or impairment in IRR with ERT in a specific outcome, *QoL* Quality of life, *SOE* Strength of evidence, *uGAGs* Urinary glycosaminoglycans.

*The SOE classification has been previously published in Bradley et .al [12]

** The analysis assessed whether the percentage of case reports showing a modification in a specific outcome was statistically higher than 5% (null hypothesis, H0). The *p*-value was performed with one-sided binomial test

*** FDR critical value: Outcomes with *p*-values lower than FDR critical value are considered as modified by ERT (Multiplicity adjustment). The FDR critical value is calculated ranking the outcomes analyzed from lowest to highest p-value. The rank (r) is divided by number of outcomes and multiplied by the accepted 10% rate of false discoveries. The lowest rank of tied *p*-values is used because the critical value computed is lower, and it is more conservative to declare a specific outcome as modified by ERT. However, the same outcomes were classified as modified by ERT when the highest or mean rank was used for ties

**** The outcomes that showed a *p*-value lower than the FDR 10% critical value were classified in the acceptable evidence group (shadow rows). The outcomes that showed a p-value higher than the FDR 10% critical value were classified in an unacceptable evidence group (white rows). In bold: Moderate to high SOE categories

The outcomes showing a *p*-value lower than the FDR 10% critical value were classified in the acceptable evidence group. These outcomes were uGAGs, liver volume and development of antibodies; they had been also classified as having moderate quality of evidence by SOE criteria in the previous meta-analysis of clinical studies. Additionally, all outcomes classified in the unacceptable evidence group (*p*-value > FDR 10% critical value) had been classified as having low to insufficient quality of evidence by the SOE criteria in the Bradley study [12].

### Sensitivity analysis of strong and weak confirmatory methods

Table 2 describes the number of case reports with a modification in a specific outcome, after ERT, as defined by the weak confirmatory method.

**Table 2:** Analysis of number of case reports showing improvement or impairment, associated with ERT in a specific outcome, based on a weak confirmatory method

RANK	Outcomes & (SOE score*)	Nr. [+] / Total cases	*p*-value**	FDR 10% Critical value***	Evidence group****
1	uGAGs **(Moderate)**	20/44	< 0.0001	< 0.009	Acceptable
2	6MWT (Low)	15/44	< 0.0001	< 0.027	Acceptable
3	Liver V **(Moderate)**	13/44	< 0.0001	< 0.027	Acceptable
4	QoL (Insufficient)	8/44	0.014	< 0.036	Acceptable
5	Antibodies **(Moderate)**	6/44	0.022	< 0.045	Acceptable
6	Growth height (Low)	5/44	0.067	N.S (> 0.055)	Unacceptable
6	JROM (Insufficient)	5/44	0.067	N.S (> 0.055)	Unacceptable
7	Pulmonary function (Low)	4/44	0.177	N.S (> 0.072)	Unacceptable
8	IRR (Low)	2/44	0.653	N.S (> 0.064)	Unacceptable
9	Sleep apnea (Insufficient)	1/44	0.895	N.S (> 0.082)	Unacceptable
10	Cardiac (Insufficient)	0/44	1	N.S (> 0.091)	Unacceptable

Legend:The improvement is defined in accordance with the weak confirmatory method. The impairment was declared when IRR caused a change in ERT dose.

*6MWT* 6-min walk test, *FDR* False discovery rate (Benjamini-Hochberg procedure), *IRR* infusion-related reaction, *JROM* joint range of motion, *Nr. [+]* Number of case reports showing improvement or impairment in IRR associated with ERT in a specific outcome, *QoL* Quality of life, *SOE* Strength of evidence, *uGAGs* Urinary glycosaminoglycans.

*The SOE classification has been previously published in Bradley et al. [12]

** The analysis assessed whether the percentage of case reports showing a modification in a specific outcome was statistically higher than 5% (null hypothesis, H0). The *p*-value was performed with a one-sided binomial test

*** FDR critical value: Outcomes with *p*-values lower than FDR critical value are considered as modified by ERT (Multiplicity adjustment). The FDR critical value is calculated by ranking the outcomes analyzed from lowest to highest p-value. The rank (r) is divided by number of outcomes and multiplied by the accepted 10% rate of false discoveries. The lowest rank of tied *p*-values was used because the critical value is computed is lower, and is more conservative for a specific outcome as modified by ERT. However, the same outcomes were classified as modified by ERT when the highest or mean rank was used for ties

**** The outcomes showing a *p-*value lower than the FDR 10% critical value were classified in acceptable evidence groups (shadow rows). The outcomes showed a p*-*value higher than the FDR 10% critical value and were classified in unacceptable evidence groups (white rows). In bold: Moderate to high SOE categories

The outcomes showing a *p*-value lower than the FDR 10% critical value were classified in the acceptable evidence group. These outcomes were uGAGs, liver volume, development of antibodies, 6MWT, and quality of live. All outcomes having moderate SOE in the previous meta-analysis were classified as acceptable in our study (uGAGs, liver volume and development of antibodies). However, there were two additional outcomes (6MWT and QoL) classified in our acceptable evidence group with low and insufficient quality of evidence by the SOE in the Bradley study [12].

In agreement with the results, outcomes classified on the strong confirmatory method show a positive predictive value (PPV), negative predictive value (NPV), sensitivity (Se), and specificity (Sp) of 100%, compared to SOE as the gold standard (Table 3). The weak confirmatory method shows lower validity indices (60% PPV, 100% NPV, 100% Se, and 75% Sp) vs. the strong confirmatory method (see Additional file 1: Table S4).

**Table 3:** Agreement between the classification of outcomes based on the case report meta-analysis and the SOE classification, based on the clinical study meta-analysis. Strong confirmatory method

	Strength of evidence of clinical study meta-analysis	
Number of case reports [+] for the outcome	High to moderate	Low to insufficient
≥ 6 [+] of 44 cases (acceptable evidence group)	(True positives = 3) -uGAGs -Liver Volume -Antibodies	(False positives = 0)
< 6 [+] of 44 cases (unacceptable evidence group)	(False negative = 0)	(True negatives = 8) -6MWT, JROM, Growth, IRR, Pulmonary function, Cardiac, QoL, sleep apnea.

Legend: The 95% confidence interval for the validity index are: positive predictive value: 100% (29 to 100%); negative predictive value: 100% (63 to 100%); sensibility: 100% (29 to 100%), and specificity: 100% (63 to 100%).

*6MWT* 6-min walk test, *CI* Confidence interval, *IRR* Infusion-related reaction, *JROM* Joint range of motion, *NPV* Negative predictive value, *PPV* Positive predictive value, *QoL* Quality of life, *Se* Sensitivity, *Sp* Specificity, *SOE* Strength of evidence, *uGAGs* Urinary glycosaminoglycans.

**Fig. 2:**
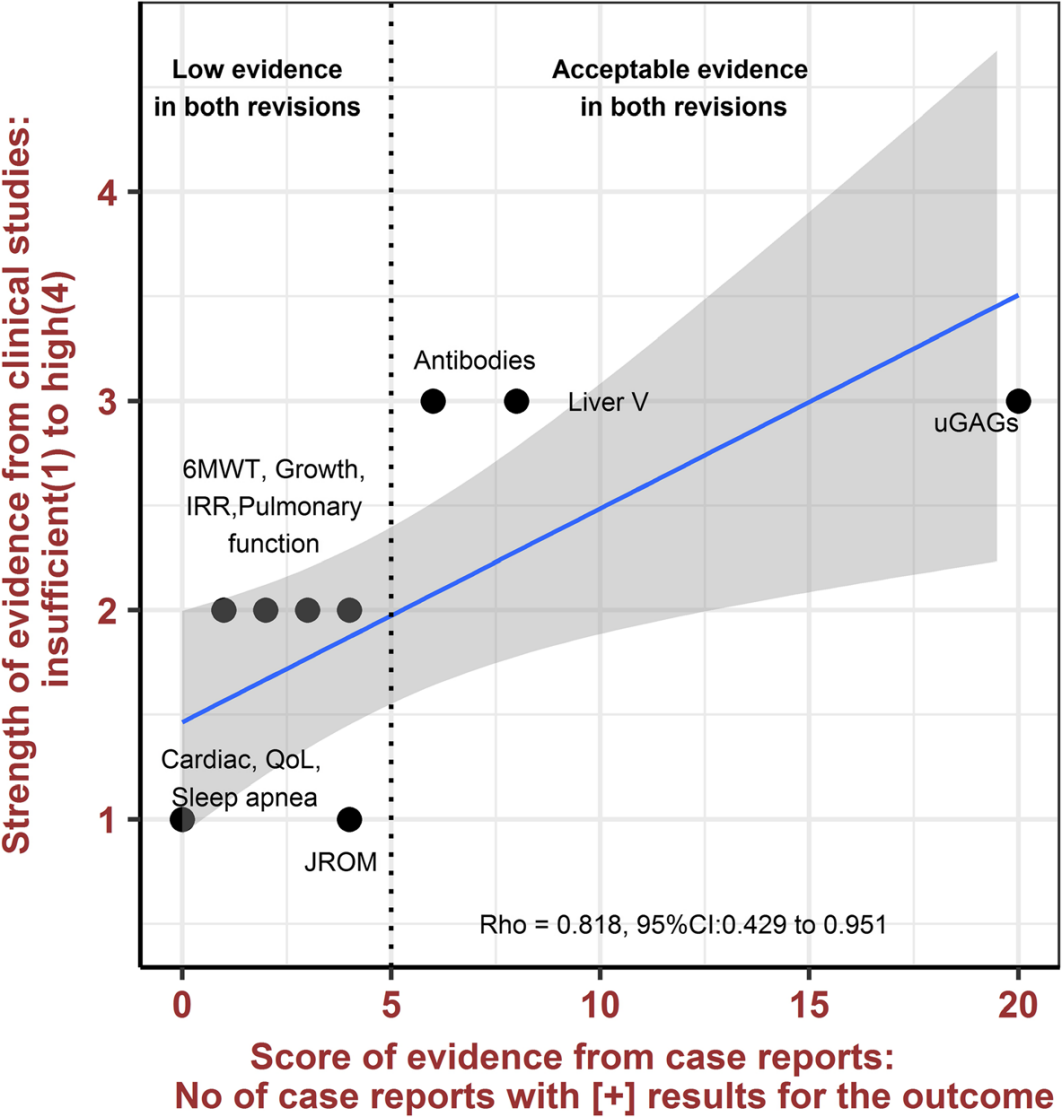
Agreement between the evidence score from the case report meta-analysis and the SOE from the clinical study meta-analysis. Strong confirmatory method. 6MWT: 6-min walk test; CI: Confidence interval; IRR: Infusion-related reaction; JROM: Joint range of motion; QoL: Quality of life; Rho: Spearman correlation coefficient; SOE: Strenght of evidence; uGAGs: Urinary glycosaminoglycans

Likewise, the relative rate of agreement between the quantitative evidence score, based on case reports with ERT-modified outcomes, and the SOE were good (Rho = 0.82, 95%CI: 0.43 to 0.95) when the strong confirmatory method was used (Fig. 2). Conversely, evaluation of ERT-modified outcomes in case reports based on the weak confirmatory method showed a moderate rate of agreement (Rho = 0.63, 95%CI: 0.044 to 0.89) with the SOE (see Additional file 1: Figure S1).

### Sensitivity analysis based on different analysis sets

The outcomes classification based on the strong confirmatory method achieved equivalent results vs. the SOE classification in the meta-analyses of clinical studies in all analysis sets [at least 10 among 11 outcomes equally classified (Accuracy ≥91%)]. In addition, the ratio of agreement between number of case reports with improved outcomes and the SOE score was good (Rho > 80%).

When we excluded congress communications from the analysis set, the accuracy between our classification (based on the strong confirmatory method) and the SOE classification was reduced to 91%, there was no detection of the development of antibodies as modified by ERT in our meta-analysis (Table 4).

**Table 4:** Sensitivity analysis based on different analysis sets

	Publications / Cases	True (+/−); False (+/−)	Accuracy%	Se%	Sp%	PPV%	NPV%	Rho (95%CI)
Primary analysis set^a^	25 / 44	(3/8); (0/0)	100	100	100	100	100	82 (43 to 95%)
All case reports^b^	38 / 56	(3/8); (0/0)	100	100	100	100	100	83 (47 to 96%)
Analyzed in Bradley study excluded^c^	22 / 33	(3/8); (0/0)	100	100	100	100	100	82 (43 to 95%)
Excluding congress communications^d^	21 / 36	(2/8); (0/1^e^)	91	67	100	100	89	82 (43 to 95%)

Legend: ^a^All case reports of male MPS-II treated with ERT report efficacy and safety. These cases reports were written in a narrative form (results not aggregated). They were published prior to Bradley bibliographic search

*6MWT* 6-min walk test, *CI* Confidence interval, *NPV* Negative predictive value, *PPV* Positive predictive value, *Rho* Spearman correlation coefficient, *Se* Sensitivity, *Sp* Specificity.

^b^All case reports of male MPS-II treated with ERT report efficacy and safety, despite being published after the bibliographic search for Bradley (31 December 2015)

^c^All case reports included in the primary analysis set, excluding all studies also analyzed in the Bradley study

^d^All case reports included in the primary analysis set, excluding all congress communications

^e^Development of antibodies was an outcome that did not show a significant modification, although it was classified as high to moderate SOE in the Bradley study.

### Sensitivity analysis based on a different null hypothesis

The best validity indices were observed with the preplanned futility boundary (5%, null hypothesis). Specificity and positive predictive values were reduced with boundaries lower than 5%. Sensitivity and negative predictive values were reduced with boundaries higher than 5% (see Additional file 1: Table S5).

## Discussion

Personalized medicine based on molecular diagnosis has fragmented complex diseases, such as cancer, into multiple molecular subtypes, each one representing a rare disease [18, 19]. This has extended the classification of rare disease to other illnesses that were not previously considered as such. Thus, the recent importance of research methods derived from rare diseases [20], the development and improvement of rare disease registries [21] and the recovered interest in case reports for aggregating results in systematic reviews [7].

There are few publications aggregating case report results in a quantitative manner [22]. There is only one study comparing the results of a case report meta-analysis and a meta-analysis including RCTs [11]; it showed that both meta-analyses reach similar conclusions in adult MPS-I.

We selected the Bradley meta-analysis [12] as the gold standard, because it analyzed the overall MPS-II population without an age restriction. Furthermore, it had been recently published and developed by an independent research group. Along with the Bradley study, the efficacy and safety of ERT in patients with MPS-II had been analyzed in three previous meta-analyses: that by da Silva et al. in 2016 [23], which only selected one phase II/III trial [24], that by Alegra et al. in 2013 [25], which combined 2 RCTs [24, 26] and 1 open label study with the same patients of all ages [27], 1 open-label study of adults [28], and 1 cohort study of children [29], plus the one by Pérez-López et al. in 2018, which analyzed adult MPS-II patients (> 16 years) [30].

In agreement with our previous study [11], we proposed a single method to aggregate results from different case reports. We considered the number of cases, showing a certain characteristic among the total number of cases analyzed. This method has the flexibility of combining outcomes independent of the measurement of the variable; it also allows different ways for controlling the multiplicity, depending on the relationship among the outcomes analyzed [17]. Additionally, we could consider the information provided from aggregated results of case reports as a single observational study and combine the results with case series, clinical trials and rare disease registries in a meta-analysis; or we could simply add the cases of all studies, as if it was a single study [22]. This allows incorporating all available evidence (single case observations, clinical studies and rare disease registries) to evaluate a particular research question. As an example, previous studies in infectious disease have used this strategy to develop classification tree models to predict disease outcomes [22].

However, the publication bias and heterogeneity of the included studies represents two critical aspects that were not considered in previous case report aggregations [13, 31]. Regarding publication bias, funnel plot tests cannot, be implemented when aggregating case reports [13]. Previous publications criticize the use of the safe-false N in meta-analyses of clinical studies [32]. This index evaluates if a significant result of a meta-analysis can become significant without considering whether the differences evaluated are clinically meaningful. This limitation can be avoided in a case report aggregation by testing if the percentage of responders is higher than the that of responders in historical controls (clinically meaningful difference). In accordance, previous clinical trials have demonstrated the utility of rare disease registries as historical controls [21].

We have proposed to analyze heterogeneity based on different sensitivity analysis to evaluate the robustness of the meta-analysis results. We would consider that an equivalent approach may be easily developed in further publications [33]. Accordingly, we have proven the robustness of our results through a specific strategy: considering all selected case reports, excluding those published after the clinical study meta-analysis bibliographic search, and excluding studies analyzed in clinical study meta-analyses, including excluding congress communications. In all scenarios, our results show good agreement with the SOE score of clinical study meta-analyses.

Some authors have underlined the utility of N-of-1 trials to compare the effect of different treatments in only one patient [34]. These designs can randomize repeated cycles of treatment challenges (e.g., A-B-A-B) in a single participant, in which A is the test drug and B is the comparison drug. These studies achieve the usual methodological safeguards of classical clinical trials (controlled, randomized, and blinded). However, these designs are not applicable in situations where the disease is not clinically stable or the carry-over effects of treatment cannot be avoided [35]. Therefore, in some diseases, most of the available evidence comes from case reports [2]. Methods to aggregate results of different N-of-1 trials in a meta-analysis assumed randomized allocation of treatment exposure about study periods [36, 37]. They cannot be applied to aggregate results of case report narratives or rare diseases registries.

As we have mentioned, rare disease registries may be valuable sources of information not only on disease course but also on treatment outcomes. A global registry, Hunter Outcome Survey (HOS), has been collecting information on patients with MPS II for over 10 years [38]. Our results seem to agree with those from the registry. Based on data from the HOS registry [39], ERT with idursulfase has a positive effect on uGAGs, and liver volume, 2 outcomes showed as of acceptable evidence in our analysis by the strong method, and also on 6MWT, which was also categorized in our analysis as acceptable evidence by the weak method. In addition, data from the HOS registry showed that 59% of patients younger than 12 years and 67% of those 12 years or older were positive for antibodies by week 13 of treatment [40], in agreement with antibody development being classified as acceptable evidence by the strong method in our analysis.

Regarding IRRs, which our analysis categorized as unacceptable evidence, we only considered as relevant the IRRs that caused ERT dose changes. Data from the HOS registry showed that although 32% of patients suffer from IRRs, most patients (85%) experience them during the first 3 months of treatment and most IRRs are mild or moderate in severity and can be managed without interrupting treatment [38].

An important point to consider is the futility boundary selected in our analysis (null hypothesis). The objective of Bradley et al. [12] and their meta-analyses was to identify benefits and harms of ERT, with the study defined as a pilot. There was not a criterion for clinical meaningfully effect. Therefore, the objective of the meta-analysis was similar to phase II designs, in which it is intended to explore the benefits and harms of a specific treatment. In accordance with previous publications evaluating treatment activity in phase II trials [41], a percentage of patients equal or lower than 5% showing a response has been considered as the null hypothesis. Accordingly, we observed that the best agreement with clinical study meta-analysis results were observed with the preplanned limit of no effect of 5%, which agrees with previous recommendations in designs with the same purpose [41]. Case report meta-analyses with other purposes, e.g., identifying effects higher than an active comparator, may require the null hypothesis to be based on historical control estimations.

We have demonstrated that standardization and a good definition of outcomes evaluated in case reports are strongly related with the validity of the results obtained based on their aggregation [8]. Thus, excluding results from poorly defined outcomes is a useful criterion to control the quality of single cases in a case report meta-analysis, as required in any meta-analysis [13].

Different authors have underlined the impact of clinical report results in clinical practice and research [1], while clinical cases have traditionally been of great importance in determining patient treatment in the context of rare diseases [7]. Our study suggests that the combination of these single cases can lead to robust results. Previous experience in a personalized medical context suggests that understanding the biologic mechanism of disease is more critical for treatment success in pivotal studies than a simple demonstration of superiority in a randomized-controlled study [39, 42].

Clinical reports have a high risk of publication bias [6] and it is expected that only positive results will be published. As a conservative assumption, we considered all outcomes not reported in a case study to not have improved. However, this assumption cannot prevent bias related to unpublished cases. Based on this issue, an alternative explanation of study results is that case reports confirming clinical study conclusions have a higher probability of being accepted and published in a journal. However, this does not explain that the level of agreement of case reports and clinical study meta-analyses results was higher, selecting only standardized and well-defined outcomes.

Another important limitation is that we cannot estimate the effect size of an outcome. Nevertheless, we observed that most clinical cases do not report enough information to aggregate study results in a mean, median, or a proportion with a confidence interval. This highlights the importance of initiatives to homogenize and upgrade the quality of the information published in case reports [9]. Additionally, we did not analyze the effect of ERT, taking into account the different treatment doses used in case reports, since we intend to compare our results with those of Bradley et al. [12], who they did not report this subgroup analysis. Either way, most cases evaluated in both studies were treated with a standard dose (0.5 mg/kg/weekly).

This analysis was confirmed in a MPS-II population treated with ERT, with results explored in a specific group of MPS-I patients. New studies must assess if results can be generalized for other diseases and patient profiles.

A meta-analysis of clinical reports cannot replace evidence provided by clinical trials. Subject recruitment in rare diseases and personalized medicine represent a critical task in clinical research [2, 43, 44]. In a therapeutic context, in which most studies become clinical reports, excluding them from systematic review increases the risk of bias and reduces efficiency, as all available evidence is not considered [45]. There is evidence that case reports translate useful data collection in cases of rare phenomena, and contribute to the progress and dissemination of novel scientific discoveries three or more years earlier than clinical studies [11]. In this period, daily clinical practice or the design of confirmatory clinical trials require evidence from published clinical reports [8].

## Conclusions

We demonstrated the agreement between the results from case reports and clinical studies based on meta-analyses, which evaluate efficacy and safety of enzyme replacement therapy in patients with MPS-II. These results confirm previous results observed in MPS-I adult patients.

We suggest that quantitatively combining results from case reports with standardized and well-defined outcomes, rather than analyzing them separately or qualitatively, may improve clinical evidence of the effect of a therapeutic strategy.

Additionally, we have proposed some methods to evaluate publication bias and heterogeneity of the included studies in a meta-analysis of case reports.

Case reports meta-analyses might help improving the clinical practice and the design of clinical trials in the context of rare diseases and increasingly in other areas of personalized medicine.

## Supplementary information

**Additional file 1: Table S1.** Search syntax used in different databases to gather the bibliographic data. **Table S2.** Case reports of males with MPS-II published prior to the bibliographic search of meta-analysis of clinical studies (January 2008 to December 2015). **Table S3.** Case reports of males with MPS-II published later to the bibliographic search of the meta-analysis of clinical studies (January 2016 to April 2018). **Table S4.** Agreement between the classification of outcomes based on the case report meta-analysis and the SOE classification based on the clinical study meta-analysis. Weak confirmatory method. **Table S5.** Sensitivity analysis on different futility boundaries. **Figure S1.** Agreement between the score of evidence from the case report meta-analysis and SOE from the clinical study meta-analysis. Weak confirmatory method.

**Additional file 2.** This file includes all the references screened in the systematic review and the reason for exclusion.

## Data Availability

All data generated or analysed during this study are included in this published article [and its supplementary information files].

## Abbreviations

6MWT: 6-min walk test
CI: Confidence interval
EMBASE: Excerpta Medica Data Base
ERT: Enzyme replacement therapy
FDR: False discovery rate (Benjamini-Hochberg procedure)
FVC: Forced vital capacity
GAG: Glycosaminoglycans
H0: Null hypothesis
IRR: Infusion-related reaction
JROM: Joint range of motion
LILACS: Latin American and Caribbean Literature on Health Sciences
MOOSE: Meta-analyses Of Observational Studies in Epidemiology
MPS-I: Mucopolysaccharidosis Type I
MPS-II: Mucopolysaccharidosis Type II
NPV: Negative predictive value
Nr. [+]: Number of case reports
PPV: Positive predictive value
PRISMA: Preferred Reporting Items for Systematic Reviews and Meta-Analyses
PROSPERO: International prospective register of systematic reviews
QoL: Quality of life
r: Rank
RCTs: Randomized clinical trials
Rho: Coeficiente de correlación de Spearman
SAE: Serious adverse event
Se: Sensitivity
SOE: Strength of evidence
Sp: Specificity
uGAGs: Urinary glycosaminoglycans
