# Supplementary material for: Agreement between results of meta-analyses from case reports and clinical studies, regarding efficacy and safety of idursulfase therapy in patients with mucopolysaccharidosis type II (MPS-II). A new tool for evidence-based medicine in rare diseases
Source: Orphanet J Rare Dis. 2019 Oct 21;14:230. doi: 10.1186/s13023-019-1202-6 (PMC6805333; doi:10.1186/s13023-019-1202-6)
Supplement: Supplementary file 1 — Additional file 1: Table S1. Search syntax used in different databases to gather the bibliographic data. Table S2. Case reports of males with MPS-II published prior to the bibliographic search of meta-analysis of clinical studies (January 2008 to December 2015). Table S3. Case reports of males with MPS-II published later to the bibliographic search of the meta-analysis of clinical studies (January 2016 to April 2018). Table S4. Agreement between the classification of outcomes based on the case report meta-analysis and the SOE classification based on the clinical study meta-analysis. Weak confirmatory method. Table S5. Sensitivity analysis on different futility boundaries. Figure S1. Agreement between the score of evidence from the case report meta-analysis and SOE from the clinical study meta-analysis. Weak confirmatory method. [file 13023_2019_1202_MOESM1_ESM.docx]

**Additional file 1:**

**Supplementary table 1. Search syntax used in different databases to gather the bibliographic data.**

| **1. Pubmed:** |
| --- |
| (("Mucopolysaccharidosis"[All Fields] AND "II"[All Fields]) OR "Mucopolysaccharidosis II"[All Fields] OR "MPS II"[All Fields] OR "Hunter syndrome"[All Fields]) AND ("idursulfase"[All Fields] OR "enzyme replacement therapy"[All Fields] OR "ert"[All Fields]) OR "iduronate-2-sulphatase"[All Fields] AND ("case reports"[Publication Type] OR "case"[All Fields] OR "report"[All Fields]) |
|  |
| **2. Embase:** |
| 'hunter syndrome' AND ('enzyme replacement' OR 'iduronate 2 sulfatase' OR 'idursulfase') AND ('case report' OR 'case study' OR 'medical record review') |
|  |
| **3. Cochrane:** |
| (“Mucopolysaccharidosis II” OR "Hunter syndrome") AND (“enzyme replacement” OR idursulfase OR “iduronate-2-sulphatase”) |
|  |
| **4. LYLACS (webpage: http://lilacs.bvsalud.org/es/):** |
| (Title, Summary, Issue) |
| “mucopolysaccharidosis II” and “'enzyme replacement” |

**Supplementary table 2. Case reports of males with MPS-II published prior to the bibliographic search of meta-analysis of clinical studies (January 2008 to December 2015).**

| **Reference**  **Publication**  **Included in Bradley study.** | **(Severe or attenuated)**  **Mutations** | **Age at diagnosis**  **Age at ERT start**  **ERT duration** | **Treatment**  **IV Dose**  **Schedule** | **Outcomes evaluated in Bradley meta-analysis modified (improved or impaired (IRR)) after ERT**  **(Novelties)** |
| --- | --- | --- | --- | --- |
| **Studies published prior to the bibliographic search of the meta-analysis of clinical studies (2008 to December 2015)** | | | | |
| **Kim et al, 2014 ^1^**  Journal article  Not included | (S)  ? | 72 months  72 months  15 months | Idursulfase  0.5 to 1  mg/kg/weekly | **uGAGs; LiverV**; 6MWT or endurance; pulmonary function; **antibodies**.  (Immune modulation protocol) |
| **NoH et al, 2014 ^2^**  Letter to editor  Not included | (?)  ? | 72 months  72 months  4 months | Idursulfase  0.5  mg/kg/weekly | (Skin lesions decrease after ERT) |
| **Lampe et al, 2014 ^3^**  Journal article  1 MPS-II  *Included* | (?)  p.R88H mutation | Pre-natal  0.3 months  2.3 months | Idursulfase  0.5  mg/kg/weekly | **uGAGs.**  (Safety and efficacy evaluation of ERT) |
| **Lampe et al, 2014 ^3^**  Journal article  1 MPS-II  *Included* | (?)  p.R95G mutation | 1 week  1.4 months  22.6 months | Idursulfase  1.5 to 0.5  mg/kg/weekly | **LiverV**  (Safety and efficacy evaluation of ERT) |
| **Lampe et al, 2014 ^3^**  Journal article  1 MPS-II  *Included* | (?)  p.P86L mutation | 6 weeks  2 months  2.3 months | Idursulfase  0.6 to 0.5  mg/kg/weekly | **uGAGs.**  (Safety and efficacy evaluation of ERT) |
| **Lampe et al, 2014 ^3^**  Journal article  *Included* | (?)  p.R493P mutation | 1 day  2.3 months  36 months | Idursulfase  0.6 to 0.5  mg/kg/weekly | **uGAGs;** LiverV; 6MWT or endurance; Growth  (Safety and efficacy evaluation of ERT) |
| **Lampe et al, 2014 ^3^**  Journal article  *Included* | (?)  c.1270insCC | 4 weeks  2.5 months  5 years | Idursulfase  0.5  mg/kg/weekly | **uGAGs;** LiverV.  (Safety and efficacy evaluation of ERT) |
| **Lampe et al, 2014 ^3^**  Journal article  *Included* | (?)  p.G336E | 11 weeks  2.8 months  17 months | Idursulfase  0.5  mg/kg/weekly | **LiverV.**  (Safety and efficacy evaluation of ERT) |
| **Lampe et al, 2014 ^3^**  Journal article  *Included* | (?)  c.1133A>G | 1 week  6 months  4 years | Idursulfase  0.5  mg/kg/weekly | (Safety and efficacy evaluation of ERT) |
| **Lampe et al, 2014 ^3^**  Journal article  *Included* | (?)  c.1362-1365dup | 5.5 months  6.5 months  4 years | Idursulfase  0.66 to 0.5  mg/kg/weekly | **uGAGs;** LiverV.  (Safety and efficacy evaluation of ERT) |
| **Christianto et al, 2013 ^4^**  Journal article  Not included | (S)  c.1053delT in exon 8 | 6 years  27 years  12 months | Idursulfase  0.5  mg/kg/weekly | **uGAGs; LiverV**; 6MWT or endurance; **antibodies**.  (Safety and efficacy evaluation of ERT) |
| **Volpi et al, 2013 ^5^**  Journal article  Not included | (S)  P120R mutation on Xq28 | 2 years and 9 m.  3 years  10 months | Idursulfase  0.5  mg/kg/weekly | **uGAGs**  (Study of plasmatic  dermatan sulfate (DS) during ERT) |
| **Sato et al, 2013 ^6^**  Journal article  Not included | (S)  ? | 3 years  7 years  24 months | Idursulfase  0.5  mg/kg/weekly | (Limited efficacy for  cardiac valve disease of ERT) |
| **Tajima et al, 2013 ^7^**  Journal article  *Included* | (S)  Recombination IDS gene and the IDS-2 pseudogene | 3 years  3 years  34 months | Idursulfase  0.3 - 0.5  mg/kg/weekly | **uGAGs; LiverV**  (Safety and efficacy evaluation of ERT) |
| **Tajima et al, 2013 ^7^**  Journal article  *Included* | (S)  Recombination IDS gene and the IDS-2 pseudogene | 4 months  4 months  32 months | Idursulfase  0.5  mg/kg/weekly | **uGAGs;**  (Safety and efficacy evaluation of pre-symptomatic initiation of ERT) |
| **Puiu M et al, 2013 ^8^**  Journal article  Not included | (S)  ? | 3 years  3 years and 3 m.  1 year | Idursulfase  0.5  mg/kg/weekly | **LiverV**; 6MWT; JROM; Growth; QoL; Sleep apnea.  (Improvement of cognitive and conductual functioning after ERT) |
| **Marín LL et al, 2012 ^9^**  Short report  Not included | (A)  ? | 6 years  9 years  9 months | Idursulfase  0.5  mg/kg/weekly | (Improvement of skin lesion after ERT) |
| **Hoffmann B et al, 2011 ^10^**  Journal article  Not included | (A)  A85T, missense mutation | 8 years  ?  20 months | Idursulfase  0.5  mg/kg/weekly | LiverV; 6MWT or endurance; **Growth;** QoL;  (Safety and efficacy evaluation of ERT) |
| **Hoffmann B et al, 2011 ^10^**  Journal article  Not included | (S)  missense  mutation C184F | 5 years  ?  22 months | Idursulfase  0.5  mg/kg/weekly | LiverV; 6MWT or endurance; **Growth;** QoL;  (Safety and efficacy evaluation of ERT) |
| **Hoffmann B et al, 2011 ^10^**  Journal article  Not included | (S)  131del10, frame-shift mutation | 5 years  ?  31 months | Idursulfase  0.5  mg/kg/weekly | 6MWT or endurance; **Growth;** QoL;  (Safety and efficacy evaluation of ERT) |
| **Tylki-Szymanska et al, 2012 ^11^**  Journal article  *Included* | (¿)  missense mutationc.1568A>G in exon 9 of the IDS gene | 3 months  3 months  36 months | Idursulfase  0.5  mg/kg/weekly | **uGAGs;**  (Safety and efficacy evaluation of ERT) |
| **Papadia F et al, 2011 ^12^**  Journal article  Not included | (S)  Splice site mutation(c.418+1  G>C). | 3 years  4 years ant 10 m. 3 years | Idursulfase  0.5  mg/kg/weekly | **uGAGs;** **LiverV; JROM**;  (Early use of ERT improve bone abnormalities) |
| **Pérez-Calvo et al, 2011**^13^  Journal article  Not included | (A)  un genotipo R443/X | 18 months  30 years  6 months | Idursulfase  0.5  mg/kg/weekly | **uGAGs; 6MWT; JROM**; QoL; **antibodies**.  (The JROM in later stages of disease may benefit from ERT) |
| **Tchan MC et al, 2011** ^14^  Journal article  Not included | (A)  ? | 20 years  44 years  12 months | Idursulfase  30  mg/weekly | **uGAGs; 6MWT;** QoL.  (Safety and efficacy evaluation of ERT in adult age) |
| **Tchan MC et al, 2011** ^14^  Journal article  Not included | (A)  ? | 26 years  51 years  12 months | Idursulfase  36  mg/weekly | **uGAGs; 6MWT**; QoL.  (Safety and efficacy evaluation of ERT in adult age) |
| **Tchan MC et al, 2011** ^14^  Journal article  Not included | (A)  ? | 22 years  46 years  12 months | Idursulfase  36  mg/weekly | **uGAGs;** QoL; **IRR.**  (Safety and efficacy evaluation of ERT in adult age) |
| **Wang RY et al, 2009**  ^15^  Journal article  Not included | (A)  homozygous P533R IDUA mutations | 3 years and 9 m.  3 years and 11 m.  2 years and 6 m. | Idursulfase  0.5  mg/kg/weekly | **uGAGs;**  (Evaluate central nervous system effects in MPS II patients) |
| **Wang RY et al, 2009**  **^15^**  Journal article  Not included | (A)  IDS mutation, hemizygous R8X mutation | 4 years and 7 m.  4 years and 11 m.  ? | Idursulfase  0.5  mg/kg/weekly | **uGAGs;**  (Evaluate central nervous system effects in MPS II patients) |
| **Galán Gómez E et al, 2008 ^16^**  Letter to editor  Not included | (S)  I2S gene showed an N350H  mutation in exon 8 | 7 months  3 years  27 weeks | Idursulfase  0.5  mg/kg/weekly | **uGAGs; Liver;** 6MWT; **antibodies**.  (The JROM in later stages of disease may benefit from ERT) |
| **Westhoff M et al, 2011 ^17^**  Journal article  Not included | (A)  ? | 3 years  37 years  24 months | Idursulfase  0.5  mg/kg/weekly | **uGAGs; 6MWT; JROM; pulmonary function;**  (ERT benefits adult Hunter patients in restrictive ventilatory defects.) |
| **Sanchez JI et al, 2015 ^18^**  Congress  Not included | ?  ? | ?  ?  ? | Idursulfase  ?  ? | (ERT improve macular edema in MPS-II patient.) |
| **Gkavogiannakis N et al, 2015 ^19^**  Congress  1 MPS-II  Males | (A)  ? | 34 years  ?  ? | Idursulfase  ?  ? | **IRR; antibodies.**  (Successful desensitization procedure to idursulfase.) |
| **Fischer et al, 2015 ^20^**  Congress  Not included | ?  ? | ?  4 years  ? | Idursulfase  ?  ? | (Idursulfase did not precipitate/worsen autoimmune anemia or thrombocytopenia) |
| **Lau HA et al, 2015 ^21^**  Congress  Not included | (A)  ? | ?  35 years  21 months | Idursulfase  ?  ? | (ERT did not prevent progression of vision loss) |
| **Kinoshita M et al, 2014 ^22^**  Congress  Not included | (A)  ? | 5 years  20 years  ? | Idursulfase  ?  ? | (ERT improves cortical function but aggravated epileptogenic.) |
| **Bivina L et al, 2014 ^23^**  Congress  Not included | ?  ? | 6 years  6 years  4 years | Idursulfase  ?  ? | (Early ERT and transplant slowed progression of the disease) |
| **Bivina L et al, 2014**  **^23^**  Congress  Not included | ?  ? | 2.5 years  2.5 years  8.5 years | Idursulfase  ?  ? | (Early ERT and transplant slowed progression of the disease) |
| **Bivina L et al, 2014**  **^23^**  Congress  Not included | ?  ? | Pre-nataly  4 months  ? | Idursulfase  ?  ? | Growth; developmental improvements  (Early ERT and transplant slowed progression of the disease) |
| **Nava E et al, 2012 ^24^**  Journal article  Not included | (S)  complete exon 7 deletion in the iduronate 2-sulfatase gene | 2 years and 4 m.  4 years and 9 m.  2 years and 1 m. | Idursulfase  ?  ? | 6MWT; **JROM;**  (Botulinum Toxin for the  Treatment of Equinus Deformity in MPS-II Patients) |
| **Nava E et al, 2012 ^24^**  Journal article  Not included | (S)  ? | 1 year and 1 m.  6 years and 6 m.  3 years | Idursulfase  ?  ? | (Botulinum Toxin for the  Treatment of Equinus Deformity in MPS-II Patients) |
| **Bonanni P et al, 2012 ^25^**  Journal article  Not included | (S)  ? | 1 year and 7 m.  8 years and 3 m.  14 months | Idursulfase  ?  ? | (Epilepsy may be a treatable cause of neurological regression in  individuals with MPS II) |
| **Uz B et al, 2012 ^26^**  Letter to editor  Not included | (A)  ? | Newborn period  10 years and 2 m.  8 months | Idursulfase  0.5  mg/kg/weekly | (Hunter syndrome and new onset idiopathic thrombocytopenic purpura) |
| **Farooq MU et al, 2008 ^27^**  Letter to editor  Not included | ?  IDS gene, a (A>T) change at nucleotide 595 | 2 year  11 years and 6 m.  12 months | Idursulfase  0.5  mg/kg/weekly | Liver; pulmonary function;  (Novel mutation in the Iduronate 2 sulfatase gene resulting in MPS-II and Chorea.) |
| **Farooq MU et al, 2008**  **^27^**  Letter to editor  Not included | ?  IDS gene, a (A>T) change at nucleotide 595 | 4 years  13 years  12 months | Idursulfase  0.5  mg/kg/weekly | Liver; pulmonary function;  (Novel mutation in the Iduronate 2 sulfatase gene resulting in MPS-II and Chorea.) |
| *^?:No data in the study´s paper; 6MWT: 6-minute walk test; Cardiac (ECHO): Cardiac evaluation with echocardiogram; IRR: infusion-related reaction; IV: Intra-venous; JROM; joint range of motion; MPS-II: Mucopolysaccharidosis type II; QoL: Quality of life; SOE: Strenght of evidence; uGAGs: Urinary glycosaminoglycans.^* | | | | |

**Supplementary table 3. Case reports of males with MPS-II published later to the bibliographic search of the meta-analysis of clinical studies (January 2016 to April 2018).**

| **Reference**  **Publication** | **(Severe or attenuated)**  **Mutations** | **Age at diagnosis**  **Age at ERT start**  **ERT duration** | **Treatment**  **IV Dose**  **Schedule** | **Outcomes evaluated in Bradley meta-analysis modified (improved or impaired (IRR)) after ERT**  **(Novelties)** |
| --- | --- | --- | --- | --- |
| **Studies published later to the bibliographic search of the meta-analysis of clinical studies (January 2016 to April 2018).** | | | | |
| **Kim et al, 2017 ^28^**  Journal article | (S)  ? | 14 months  15 months  5 years | Idursulfase  0.5 to 1 mg/kg/weekly | **uGAGs;** 6MWT or endurance; JROM; pulmonary function; **antibodies**.  (uGAGs as biomarker for antibodies;  Anti-immunological scheme) |
| **Ngu et al, 2017 ^29^**  Journal article | (A)  c.1608_1609delTA (p.Tyr536Ter) mutation  exon 9 IDS gene | 6 years  11 years  20 / 24 months | Idursulfase / idursulfase beta  0.5 / 1.67 to 0.5  mg/kg/weekly | **uGAGs; LiverV**; **6MWT**; growth; **Cardiac (ECHO);** sleep disorder; **antibodies**.  (idursulfase beta after idursulfase as  Anti-immunological scheme) |
| **Nishiyama et al, 2016 ^30^**  Journal article | (A)  ? | 6 years  6 years  18 months | Idursulfase  0.5  mg/kg/weekly | **uGAGs;** LiverV; Spleen Volume; JROM; sleep disorder.  (Hydroneprhosis resolution) |
| **Gupta et al, 2014 ^31^ &**  **Madireddi et al, 2016 ^32^**  Journal article | (A)  mutation A85T  caused by a G to A substitution at nucleotide position c.253 in the exon 3 of IDS | 24 years  24 years  4 months | Idursulfase  0.5  mg/kg/weekly | **Spleen Volume; 6MWT;** JROM; pulmonary function; QoL.  (Diagnosis of MPS-II by enzyme  assay and mutational analysis) |
| **Akiyama R et al, 2018 ^33^**  Congress | (A)  ? | 12 years  12 years  ? months | Enzyme replacement therapy | Growth  (Optic abnormalities not changed by ERT treatment) |
| **Al B et al, 2017 ^34^**  Journal article | (S)  ? | 10 days  10 days  1.4 months | Idursulfase  0.5  mg/kg/weekly | **uGAGs.**  (hematopoietic  stem cell transplantation (HSCT)) |
| **Jarstad A eta al, 2017** ^35^  Congress | (A)  ? | 35 years  39 years  4 years | Enzyme replacement therapy | (Optic abnormalities not changed by ERT) |
| **Moreno KJ et al, 2017 ^36^**  Congress | (A)  hemizygous mutation in intron 5 of the IDS gene, c.709-658GN A. | 25 years  25 years  6 months | Idursulfase  0.5  mg/kg/weekly | **Cardiac (ECHO)**; QoL.  (Cardiac improvement after ERT) |
| **Bettocchi I et al, 2016 ^37^**  Congress | (S)  IDS gene deletion of exons 1-7, extending to regions Xq28 e Xq27.3, removing the entire pseudogene IDS2 and genes FMR1 and AFF2 | 3 months  18 months  35. years | Idursulfase  0.5  mg/kg/weekly | (MPS-II mutation analysis) |
| **Romero FHC et al, 2016 ^38^**  Congress | (S)  IDS/IDSP1 inversion | 3 years  ? months  3 years | Idursulfase  0.5  mg/kg/weekly | **IRR.**  (Adverse events under Idursulfase treatment) |
| **Romero FHC et al, 2016 ^38^**  Congress | (S)  IDS/IDSP1 inversion | 36 months  ? months  2 years | Idursulfase  0.5  mg/kg/weekly | **IRR.**  (Adverse events under Idursulfase treatment) |
| **Romero FHC et al, 2016 ^38^**  Congress | (S)  IDS/IDSP1 inversion | 3 years  ? months  5 years | Idursulfase  0.5  mg/kg/weekly | **IRR.**  (Adverse events under Idursulfase treatment) |
| *^?:No data in the study´s paper; 6MWT: 6-minute walk test; Cardiac (ECHO): Cardiac evaluation with echocardiogram; IRR: infusion-related reaction; IV: Intra-venous; JROM; joint range of motion; MPS-II: Mucopolysaccharidosis type II; QoL: Quality of life; SOE: strenght of evidence; uGAGs: Urinary glycosaminoglycans.^* | | | | |

**Supplementary table 4. Agreement between the classification of outcomes based on the case report meta-analysis and the SOE classification based on the clinical study meta-analysis. Weak confirmatory method.**

|  | **Strength of evidence of clinical study meta-analysis** | |
| --- | --- | --- |
| **Number of case reports [+] for the outcome** | **High to  moderate** | **Low to  insufficient** |
| **≥ 6 [+] of 44 cases**  **(acceptable evidence group)** | (True positives= 3)  -uGAGs -Liver Volume -Antibodies | (False positive=2)  -6WMT, QoL, |
| **< 6 [+] of 44 cases**  **(unacceptable evidence group)** | (False negative=0) | (True negatives=6) - Growth, JROM, Pulmonary function, IRR, sleep apnea, Cardiac. |

*^The 95% confidence interval for the validity index are: positive predictive value: 60% (15 to 95%); negative predictive value: 100% (54 to 100%); sensibility: 100% (29 to 100%) and specificity: 75% (35 to 97%).^*

*^6MWT: 6-minute walk test; CI: Confidence interval; IRR: Infusion-related reaction; JROM; Joint range of motion; NPV: Negative predictive value; PPV: Positive predictive value; QoL: Quality of life; Se: Sensitivity; Sp: Specificity; SOE: Strenght of evidence; uGAGs: Urinary glycosaminoglycans.^*

**Supplementary figure 1. Agreement between the score of evidence from the case report meta-analysis and SOE from the clinical study meta-analysis. Weak confirmatory method.**


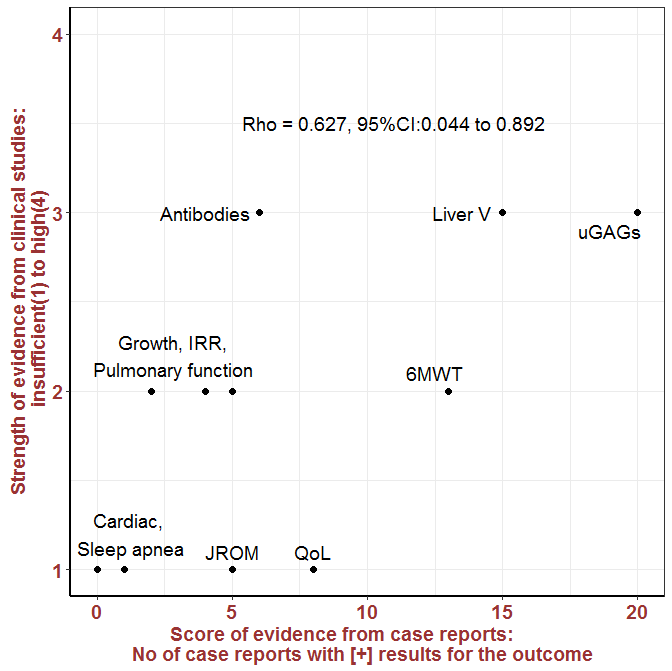


*^6MWT: 6-minute walk test; CI: Confidence interval; IRR: Infusion-related reaction; JROM: Joint range of motion; QoL: Quality of life; Rho: Spearman correlation coefficient; SOE: Strenght of evidence; uGAGs: Urinary glycosaminoglycans.^*

**Supplementary table 5. Sensitivity analysis on different futility boundaries.**

| **Futility boundary*** | **True (+/-); False (+/-)** | **Accuracy%** | **Se%** | **Sp%** | **PPV%** | **NPV%** |
| --- | --- | --- | --- | --- | --- | --- |
| 5% ** | (3/8); (0/0) | 100 | 100 | 100 | 100 | 100 |
| 1% | (3/5); (3/0) | 73 | 100 | 62 | 50 | 100 |
| 10% | (1/8); (0/2) | 82 | 33 | 100 | 100 | 80 |
| 15% | (1/8); (0/2) | 82 | 33 | 100 | 100 | 80 |
| 20% | (1/8); (0/2) | 82 | 33 | 100 | 100 | 80 |
| 50% | (0/8); (0/3) | 73 | 0 | 100 | 0 | 73 |

*^*The analyses were done in primary analysis set: All case reports of males MPS-II treated with ERT that report efficacy and safety. This case reports were written in a narrative form (results not aggregated) and published prior to Bradley bibliographic search.^*

*^** The futility boundary has been considered the null hypothesis of the analysis.^*

*^6MWT: 6-minute walk test; CI: Confidence interval; NPV: Negative predictive value; PPV: Positive predictive value; Rho: Spearman correlation coefficient; Se: Sensitivity; Sp: Specificity.^*

**REFERENCES**

1. Kim KH, Messinger YH, Burton BK. Successful reduction of high-sustained anti-idursulfase antibody titers by immune modulation therapy in a patient with severe mucopolysaccharidosis type II. *Mol Genet Metab Rep.* 2015;2:20-24.

2. Noh TK, Han JS, Won CH, et al. Characteristic "pebbling" skin eruption as a presenting sign of Hunter syndrome. *Int J Dermatol.* 2014;53(12):e594-596.

3. Lampe C, Atherton A, Burton BK, et al. Enzyme Replacement Therapy in Mucopolysaccharidosis II Patients Under 1 Year of Age. *JIMD Rep.* 2014;14:99-113.

4. Christianto A, Watanabe H, Nakajima T, Inazu T. Idursulfase enzyme replacement therapy in an adult patient with severe Hunter syndrome having a novel mutation of iduronate-2-sulfatase gene. *Clin Chim Acta.* 2013;423:66-68.

5. Volpi N, Zampini L, Maccari F, et al. Plasmatic kinetics of dermatan sulfate during enzyme replacement therapy with iduronate-2-sulfatase in a mucopolysaccharidosis II patient. *Glycoconj J.* 2013;30(7):727-732.

6. Sato Y, Fujiwara M, Kobayashi H, Ida H. Massive accumulation of glycosaminoglycans in the aortic valve of a patient with Hunter syndrome during enzyme replacement therapy. *Pediatr Cardiol.* 2013;34(8):2077-2079.

7. Tajima G, Sakura N, Kosuga M, Okuyama T, Kobayashi M. Effects of idursulfase enzyme replacement therapy for Mucopolysaccharidosis type II when started in early infancy: comparison in two siblings. *Mol Genet Metab.* 2013;108(3):172-177.

8. Puiu M, Chirita-Emandi A, Dumitriu S, Arghirescu S. Hunter syndrome follow-up after 1 year of enzyme-replacement therapy. *BMJ Case Rep.* 2013;2013.

9. Marín LL, Gutiérrez-Solana LG, Fernández AT. Hunter syndrome: resolution of extensive typical skin lesions after 9 months of enzyme replacement therapy with idursulfase. *Pediatr Dermatol.* 2012;29(3):369-370.

10. Hoffmann B, Schulze-Frenking G, Al-Sawaf S, Beck M, Mayatepek E. Hunter disease before and during enzyme replacement therapy. *Pediatr Neurol.* 2011;45(3):181-184.

11. Tylki-Szymanska A, Jurecka A, Zuber Z, Rozdzynska A, Marucha J, Czartoryska B. Enzyme replacement therapy for mucopolysaccharidosis II from 3 months of age: a 3-year follow-up. *Acta Paediatr.* 2012;101(1):e42-47.

12. Papadia F, Lozupone MS, Gaeta A, Capodiferro D, Lacalendola G. Long-term enzyme replacement therapy in a severe case of mucopolysaccharidosis type II (Hunter syndrome). *Eur Rev Med Pharmacol Sci.* 2011;15(3):253-258.

13. Pérez-Calvo J, Bergua Sanclemente I, López Moreno MJ, Torralba Cabeza M, Amores Arriaga B. [Early response to idursulfase in a 31-year old male patient with Hunter syndrome]. *Rev Clin Esp.* 2011;211(7):e42-45.

14. Tchan MC, Devine KT, Sillence DO. Three Adult Siblings with Mucopolysaccharidosis Type II (Hunter Syndrome): A Report on Clinical Heterogeneity and 12 Months of Therapy with Idursulfase. *JIMD Rep.* 2011;1:57-64.

15. Wang RY, Cambray-Forker EJ, Ohanian K, et al. Treatment reduces or stabilizes brain imaging abnormalities in patients with MPS I and II. *Mol Genet Metab.* 2009;98(4):406-411.

16. Galán-Gómez E, Guerrero-Rico A, Cáceres-Marzal C, et al. Early response to idursulfase treatment in a 3 year-old boy affected of Hunter syndrome. *Eur J Med Genet.* 2008;51(3):268-271.

17. Westhoff M, Litterst P. Successful noninvasive ventilation and enzyme replacement therapy in an adult patient with morbus hunter. *JIMD Rep.* 2012;5:77-82.

18. Sanchez JI, Ascaso FJ, Perez I, et al. Role of SD-OCT in the follow-up of a patient with macular edema associated with mucopoysaccharidosis type II (Hunter syndrome) undergoingidursulfase enzyme replacement therapy.*Acta Ophthalmologica.* 2015;93.

19. Gkavogiannakis N, Aggelides X, Makris M. Case of infusion reaction to idursulafase and successful re-administration with desensitization. *European Journal of Allergy and Clinical Immunology.* 2015;70:1.

20. Fischer A, Fernandez K, Flores J, Deshpande G, Croke B, Antony R. Autoimmune thrombocytopenia in a patient with hunter syndrome: Should iduronate-2-sulfatase replacement therapy still be considered? *Pediatric Blood and Cancer.* 2015;62.

21. Lau HA NR, Narayana K, Rucker J, Balcer L and Galetta S. Multiplemechanisms of ophthalmologic involvement in attenuated Hunter syndrome: A case report. *Molecular genetics and metabolism.* 2015;114(2):S69.

22. Kinoshita M, Furujo M, Kubo T. EEG evaluation of mucopolysaccharidosis type II after enzyme replacement therapy. *Clinical Neurology.* 2014;54.

23. Bivina L, Boyadjiev SA. Mucopolysaccharidosis type II (MPS II): case report of three affected siblings. *Molecular Genetics and Metabolism.* 2014;111(s27).

24. Nava E, Weber P, Gautschi M, Nuoffer J, Grunt S. Botulinum toxin type A for the t reatment of equinus deformity in patients with mucopolysaccharidosis yype II. *Journal of Child Neurology.* 2012;27(12):5.

25. Bonanni P, Gubernale M, Martinez F, et al. Non-convulsive status epilepticus of frontal origin in mucopolysaccharidosis type II successfully treated with ethosuximide. *Dev Med Child Neurol.* 2012;54(10):961-964.

26. Uz B, Demiroglu H, Ozcebe OI. Hunter syndrome and new onset idiopathic thrombocytopenic purpura in a young patient. *Ann Hematol.* 2012;91(2):303-304.

27. Farooq MU, Balmer SV, DeRoos ST, Houtman KL, Chillag KL. A novel mutation in the iduronate 2 sulfatase gene resulting in mucopolysaccharidosis type II and chorea: case report of two siblings. *Mov Disord.* 2008;23(10):1487-1488.

28. Kim S, Whitley CB, Jarnes Utz JR. Correlation between urinary GAG and anti-idursulfase ERT neutralizing antibodies during treatment with NICIT immune tolerance regimen: A case report. *Mol Genet Metab.* 2017;122(1-2):92-99.

29. Ngu LH, Ong Peitee W, Leong HY, Chew HB. Case report of treatment experience with idursulfase beta (Hunterase) in an adolescent patient with MPS II. *Mol Genet Metab Rep.* 2017;12:28-32.

30. Nishiyama K, Imai T, Ohkubo K, Sanefuji M, Takada H. Resolution of Hydronephrosis in a Patient With Mucopolysaccharidosis Type II With Enzyme Replacement Therapy. *Urology.* 2017;101:163-165.

31. Gupta A, Uttarilli A, Dalal A, Girisha KM. Hunter syndrome with late age of presentation: clinical description of a case and review of the literature. *BMJ Case Rep.* 2015;2015.

32. Madireddi J, P S, Shetty RK, Prabhu M, K M G. Hunter syndrome with its typical heart: a close mimic to rheumatic heart. *BMJ Case Rep.* 2015;2015.

33. Akiyama R, Murakami Y, Sengoku A, Sonoda K-H. A case of mucopolysaccharidosis (MPS) II diagnosed from the appearance of optic nerve head swelling. *Neuro-Ophthalmology Japan.* 2018;35(1):4.

34. Barth AL, de Magalhães TSPC, Reis ABR, et al. Early hematopoietic stem cell transplantation in a patient with severe mucopolysaccharidosis II: A 7 years follow-up. *Mol Genet Metab Rep.* 2017;12:62-68.

35. Jarstad A, Meeker A, Ko M. Ophthalmic findings in a hunter syndrome patient on enzyme replacement therapy. *Neurology.* 2017;88(16).

36. Moreno LJ, Sanchez-Gomez A, Satizabal JM. Short term impact from enzyme replacement therapy on patients with attenuated Hunter syndrome (MPS II) showing complex heart disease. *Molecular Genetics and Metabolism.* 2017;120(1-2).

37. Bettocchi I, Ortolano R, Baronio F, Bertola F, Pession A, Cassio A. An atypical patient with Hunter syndrome. *Journal of Inherited Metabolic Disease.* 2016;39(S266).

38. Romero FHC, Martinez JIN, Barragan DEC, Escobar IG. Severe adverse reaction to idursulfase in 3 Mexican brothers with MPS-II: Case series. *Allergy: European Journal of Allergy and Clinical Immunology.* 2016;71:1.
